# Quantification of left ventricular regional myocardial function using MRI feature tracking in healthy children - a dual-center study

**DOI:** 10.1186/1532-429X-15-S1-O42

**Published:** 2013-01-30

**Authors:** Joachim G Eichhorn, Sebastian Buss, Angela Foell, Astrid Helling, Daniel Robbers-Visser, E Pieterman, Sjoerd Bossers, Dirk Lossnitzer, Matthias Gorenflo, Willem A Helbing

**Affiliations:** 1 Pediatric Cardiology / Congenital Heart Disease, University Children's Hospital, Heidelberg, Germany; 2 Cardiology, University Hospital of Internal Medicine, Heidelberg, Germany; 3 PediatricCardiology and Radiology, Erasmus MC-Sophia children's hospital, Rotterdam, the Netherlands

## Background

Similar to speckle tracking echocardiography cardiac MR (CMR) feature tracking imaging (FTI) uses a 2-dimensional deforming analysis of the myocardium which allows quantitative evaluation of myocardial motion and contractility. This technique might provide important additional information in children, including those with congenital heart disease (CHD). Until now, there are no reference data from a healthy pediatric population. The availability of this type of data is a requirement for proper interpretation in pediatric heart disease. The aim of this study was to determine normal values of left ventricular (LV) longitudinal, circumferential and radial strain and strain rate in healthy children, using FTI in cardiovascular MR images.

## Methods

Eighty children from 2 centers (mean age: 12±5.5 years, range: 1 to 17 years; 39 female; divided into 3 groups: 0-11y (n=24), 11-15y (n=32), 15-18y (n=24)) were examined at two different 1.5 T scanners (Siemens and GE) using a standard SSFP MRI protocol. LV longitudinal, circumferential and radial strain and strain rate were measured using FTI (2D CPA MR©, TomTec Imaging System, Fulda, Germany). FTI was applied to standard cine-images in the following orientations:3 LV short-axis slices (basal, middle, apical), 4- and 3-chamber views. The effects of age, gender, endo- and epicardial assessment, myocardial segments on strain and strain rate data were also evaluated.

## Results

The global longitudinal and circumferential strains (strain rate) were -18.9±4.3% (1.9±0.5) and -22.4±4.2% (2.0±0.6), respectively with higher values being observed at basal than at apical segments. Radial strain ranged from 28.6±9.3% (-2.1±0.5). For this parameter a decrease towards the apical segments was noted. Strain and strain rate measurements using FTI varied between endo- and epicardial assessment also, with regional differences (apical versus middle and basal), but not with age or gender. Strains measured on data acquired with 2 MRI scanners had good correlations. Inter- and intra-observer variability were low and acceptable (< 10%).

## Conclusions

This study provides for the first time normal FTI values of LV function in a large cohort of healthy children. FTI offers additional data about regional and global cardiac function, using routinely acquired standard SSFP sequences and standard image orientations. FTI is feasible and provides fast and reproducible quantification of global and regional LV function. The FTI technique may prove helpful in the assessment of LV function in children with CHD and may provide additional outcome markers.

## Funding

No funding.

